# Realization of ultrastrong coupling between LSPR and Fabry–Pérot mode via self-assembly of Au-NPs on p-NiO/Au film

**DOI:** 10.1515/nanoph-2023-0763

**Published:** 2024-01-19

**Authors:** Alexis Angelo R. Garcia, Cheng-An Mao, Wen-Hui (Sophia) Cheng

**Affiliations:** Department of Materials Science and Engineering, National Cheng Kung University, Tainan, Taiwan; Hierarchical Green-Energy Materials (Hi-GEM) Research Center, National Cheng Kung University, Tainan, Taiwan

**Keywords:** ultrastrong coupling, LSPR, Fabry–Pérot, Rabi splitting energy, air–liquid interface self-assembly

## Abstract

The realization of higher coupling strengths between coupled resonant modes enables exploration of compelling phenomena in diverse fields of physics and chemistry. In this study, we focus on the modal coupling between localized surface plasmon resonance (LSPR) of Au nanoparticles (Au-NPs) and Fabry–Pérot mode (p-NiO/Au film). The effects of nanoparticle size, projected surface coverage (PSC), interparticle distance (IPD), and arrangement to the coupling strength between the two modes are theoretically investigated using finite-difference time-domain (FDTD) method. Au-NPs/p-NiO/Au film (ANA) nanostructures with NPs size of 10 nm, 30 nm, and 50 nm are considered. Numerical calculations point to larger size and higher projected surface coverage (also smaller IPD) of NPs as pre-eminent factors in enhancing the strength of modal coupling. ANA nanostructure with NPs size of 30 nm (ANA-30) and 50 nm (ANA-50) are experimentally fabricated via a facile air–liquid interface self-assembly. The fabricated nanodevices exhibit immense Rabi splitting energies of 655 meV (ANA-30) and 770 meV (ANA-50), and thus fulfill the ultrastrong coupling condition with remarkable splitting energy to bare (plasmon) energy ratio of 0.35 (ANA-30) and 0.4 (ANA-50). The physical insights presented in this study, together with the simple and scalable fabrication process, establish a viable approach to realize stronger coupling between LSPR and Fabry–Pérot mode in metal NPs/dielectric/metal film systems. This will be vital to take advantage of the promising performance enhancements of plasmonic-based nanostructures under strongly coupled regimes in areas such as solar to fuel conversion, sensing, opto-electronics, and quantum applications.

## 1 Introduction

Enhancing light–matter interaction offers a powerful way to modify physical properties of materials and thus has gathered substantial research interest in photonics, chemistry, opto-electronics, nonlinear quantum optics, and a lot more. This is explored experimentally in coupling between energy transitions and confined electromagnetic field such as the exciton–plasmon coupling in semiconductor–metal heterostructures and ensembles of organic molecules within the immediate vicinity of metallic nanostructures [1], [2]. Modal coupling between resonant modes like plasmon modes and nanophotonic modes is also recently given attention [3]. The extent of interaction, generally classified as weak coupling and strong coupling, depends on rate of energy exchange between the individual subsystems relative to their average rate of energy decay in the coupled system [4]. Under the weak coupling regime, the coupling strength is not strong enough to dominate over the average losses in the system. This results to no significant shift in the resonance position but a substantial change in the dynamics of spontaneous emission rate of an energy or molecular transition is observed, widely known as the Purcell effect [5]. When the coupling strength is further increased such that the coherent exchange of energy between two coupled modes overwhelms the effective decay rates in the coupled system, the strong coupling regime is realized. An important characteristic of strong coupling is the anticrossing behavior that leads to the formation of two new light–matter hybrid states, which are separated energetically by a Rabi splitting [6]. From a two-level Hamiltonian, the energies of the two hybrid polaritons in a strongly coupled system between LSPR and a Fabry–Pérot mode can be expressed as [7]:

$$\begin{array}{ll} E_{\pm } & =\hbar \omega_{\pm }\\ & =\frac{E_{\text{LSPR}}+E_{\text{FP}}}{2}\pm \frac{1}{2}\sqrt{{\left(E_{\text{LSPR}}-E_{\text{FP}}\right)}^{2}+{\left|\hbar \Omega \right|}^{2}} \end{array}$$

where *E*_+_ is the energy of the upper polariton branch (UPB) and *E*_−_ is the energy of the lower polariton branch (LPB). The Rabi splitting energy (*ℏ*Ω = *ℏω*_+_ − *ℏω*_−_ = 2*ℏg*, where *g* is the coupling strength) is obtained when the resonance energy of the LSPR mode and Fabry–Pérot mode are equal (i.e., *E*_LSPR_ = *E*_FP_). There are instances, however, that the splitting energy is smaller than the linewidth of the resonances, which causes the hybrid peaks to overlap and, hence, no visible splitting is observed [8]. Also, other factors such as induced transparency can cause band splitting even in weak coupling regime [9]. Therefore, a stricter condition for the realization of the strong coupling regime is that the splitting energy must be larger than the linewidths of the coupled states, that is, 2*g* > [*γ*_UPB_ + *γ*_LPB_]/2, where *γ*_UPB_ and *γ*_LPB_ represent the decay of the hybrid states, which can be obtained by spectral fitting of the resonances [6], [10]. This strong coupling criterium will be considered throughout this study. When the strength of light–matter interaction reaches a point where the coupling strength, *g*, is large enough to reach a considerable fraction of the bare resonance frequency of the uncoupled subsystems, *ω*_0_, the coupling is said to be in the ultrastrong coupling regime. This condition can be expressed as *g*/*ℏω*_0_ > 0.1, or in terms of the splitting energy, 2*g* > 0.2 (*ℏω*_0_) [11].

The exploration of the strong coupling regime has opened very interesting phenomena such as enhancement of nonradiative energy transfer between molecules placed in a metal Fabry–Pérot cavity [12], modification of chemical reactivity of molecules within a resonant cavity [13], formation of Bose–Einstein condensate (BEC) for polariton lasing [14], and so on. Likewise, the ultrastrong coupling regime is of fundamental interest in a lot of studies because it allows observation of new frontiers in quantum information processing, nonlinear optics, and photochemistry [15], [16]. In subwavelength metallic materials, the LSPR effectively serves to confine and concentrate the light in nanoscale volume, accompanied with localized enhancement of the electromagnetic field surrounding the metal, making them powerful platform to experimentally study strong light–matter interactions [1], [17], [18]. Recent studies show that the strong coupling of plasmonic mode to a nanophotonic mode leads to dual band absorption covering broad spectral range that is beneficial for solar energy harvesting [19] and also facilitates ultrafast hot carrier transfer in metal/dielectric systems [20], [21]. Thus, this regime effectively addresses the two major challenges associated with plasmon-assisted photocatalysis, specifically the limited absorption of monolayer of metallic nanoparticles and the inefficient hot carrier separation and utilization due to the ultrashort lifetime of hot carriers [22]. Photochemical transformations performed under strong coupling conditions such as water splitting [23], selective fixation of dinitrogen to ammonia [24], and hydrogen evolution reaction [25] show significant boost in overall performance compared to conventional device without strong coupling. In addition, a larger coupling strength between LSPR and Fabry–Pérot mode associated ultrastrong coupling regime is shown to yield even greater quantum yield enhancements [26]. It is noted that in all these studies, the metallic nanoparticles are partially embedded into the dielectric layer to enhance the coupling between the LSPR and Fabry–Pérot mode. Insights to maximize the coupling interaction between these two modes are, therefore, important.

In light of the promising performance enhancement in nanodevices under strong and ultrastrong coupling regimes, this study aims to provide relevant physical insights on the influence of parameters such as the nanoparticle size, projected surface coverage, and its arrangement in the realization of stronger coupling interaction between LSPR mode and Fabry–Pérot mode in metallic NPs–dielectric–metal reflector (MDM) nanostructures through conducting numerical calculations using FDTD method. We believe these parameters significantly affect the strength of coupling between the two modes and knowledge of such will be vital to realize higher coupling strengths. Moreover, a facile experimental fabrication process of nanodevices conforming to the gathered insights will be demonstrated followed by validation of its coupling regime by optical measurements and Lorentzian spectral fitting. A plasmonic nanostructure consisting of spherical Au-NPs decorated on top of a p-NiO/Au film will be in focus, leading to the final ANA structure. The NPs size will be varied from 10 nm, 30 nm, and 50 nm. Periodic square lattice arrangement and hexagonal close-packed arrangement of Au-NPs will be taken into consideration. For the square lattice structures, the projected surface coverage is varied, while for the hexagonal close-packed structures, the influence of interparticle distance between the metallic will be explored to infer the onset of strong coupling in the nanostructures. Overall, the insights gathered on this study will be valuable in the design of MDM nanostructures capable of reaching extremely large splitting energies (stronger coupling interaction). This will be indispensable to take advantage of the promising enhancement in the performance of related nanodevices under the strongly coupled regimes.

## 2 Experimental section

### 2.1 Numerical simulations

To investigate the strong coupling behavior of the proposed ANA nanostructures, FDTD simulations were conducted using the commercial software packages of FDTD solutions (Lumerical, Inc.). The schematic of the systems under study for the modal strong coupling between LSPR mode and a Fabry–Pérot cavity mode is shown in Supporting Information (Figure S1). The model consists of a monolayer of spherical metallic NPs decorated on top of p-NiO/Au reflector film. The energy of the Fabry–Pérot mode will be matched to the LSPR energy by sweeping the simulation model at different p-NiO thickness to reach the optimum condition satisfying the strong coupling regime. The size effect of the nanoparticle in achieving the strong coupling regime is then probed by varying the diameter of the Au-NPs to 10 nm, 30 nm, and 50 nm. The arrangement of the nanoparticles on top of the Fabry–Pérot mode is also varied from a periodic array represented by a square lattice unit cell (SL) and hexagonal close-packed array represented by rectangular unit cell (HL-CP). Periodic boundary conditions are applied in the *x* and *y* directions, while a perfectly matched layer boundary is applied in the *z*-direction. For the square lattice arrangement, the effect of PSC is studied by varying the simulation pitch, that is, the *P*_*x*_ and *P*_*y*_ dimension of the simulation unit cell. For the HL-CP arrangement, the IPD is varied to determine the onset of the strong coupling regime and the transition between coupling regimes.

To ensure accuracy of the simulation results, mesh-override regions are set-up around the metal NPs. Convergence tests are performed with different mesh sizes until the appropriate conditions are determined. The final mesh-override sizes are as follows: 10 nm Au-NP, 0.10 nm; 30 nm Au-NP, 0.25 nm; 50 nm Au-NP, 0.35 nm. One power monitor is placed behind the source to obtain the reflection data, while another power monitor is positioned at the interface of Au-film and Si for the transmission data. The absorbance is then calculated as *A* = 1 – *T* – *R*. A plane-wave source is utilized for the simulations, while frequency-domain field and power monitors are used to acquire electric field (*E*) and magnetic field (*H*) profiles. The dielectric functions of gold are taken from the CRC Handbook of Chemistry and Physics while that of p-NiO are obtained by experimental measurement and included in the Supporting Information (Figure S2). All the simulation data including absorption spectra and electromagnetic field distributions are calculated with the FDTD solutions software.

### 2.2 p-NiO/Au film substrate preparation

Silicon wafer substrate is sequentially rinsed with deionized water, acetone, and isopropyl alcohol using an ultrasonic bath for 10 min each solvent and then dried under pure nitrogen flow. To improve adhesion of the reflective Au film, a 10 nm Cr adhesion layer is first deposited on the surface of the Si wafer and followed by the deposition of 100 nm Au film by thermal evaporation (Thermal Evaporator, Kao Duen Tech. Corp) at a deposition rate of ∼1.0 Å s^−1^. Then, the nickel oxide (NiO) thin film is sputtered onto the Au film using W-29G10 Co-sputter & thermal system (Kao Duen Tech. Corp) with an argon flow of 30 sccm, an RF power of 60 W, and deposition rate of 0.17 Å s^−1^.

### 2.3 Phase transfer of Au-NPs from aqueous to organic solvent

The colloidal dispersion of gold nanoparticles stabilized in water (Au-NPs average diameter: 30–50 nm, average concentration: 3.51 × 10^10^–1.79 × 10^11^ particles mL^−1^) is purchased from Sigma-Aldrich. To facilitate the self-assembly of Au-NPs monolayers at the air–water interface, the nanoparticles are first transferred to chloroform (CHCl_3_) solvent. This is done by using a modified solvent exchange method for plasmonic nanoparticles reported in literature [27]. Briefly, an appropriate amount of poly(ethyleneglycol) methyl ether thiol (PEG-SH, MW: 6000 g mol^−1^) is first added to the aqueous dispersion of the nanoparticles as a prestabilizer in order to prevent the aggregation of NPs during the solvent transfer process. A sufficient amount of 1-dodecanethiol (DDT, MW: 202.4 g mol^−1^) is added to the chloroform. Both the solutions are allowed to stabilize for 2 h. Then, the aqueous phase containing the Au-NPs is placed on a sample vial together with the CHCl_3_ containing DDT, forming two separate phases. The sample is subjected to vortex mixing and the phase transfer is aided by addition of 10 μL of concentrated HCl. Vigorous mixing for 5 min is enough to transfer the Au-NPs from aqueous to CHCl_3_ phase. After the phase transfer, the organic dispersion is purified and washed by centrifugation to remove excess PEG-SH and DDT. Ethanol is also added to assist the precipitation of the Au-NPs.

### 2.4 Fabrication of Au-NPs/p-NiO/Au film (ANA) heterostructure via air–liquid self-assembly

After the final washing, the supernatant liquid is removed and a suitable amount of CHCl_3_ is added to the Au-NPs to obtain a concentrated dispersion. It is then immersed in an ultrasonic bath for 10 s to ensure that the Au-NPs are well dispersed before it is spread on top of the water subphase. The final dispersion is carefully spread out on top of the subphase by dropwise addition until the dispersion completely covered the water surface. The organic solvent is then allowed to evaporate, leaving the monolayer of Au-NPs on top of the air/water interface. The monolayer is transferred onto the previously prepared substrate by gently touching the substrate on the self-assembled monolayer.

### 2.5 Characterization

Thin film thickness measurements are done by ellipsometry using M2000-DI ellipsometer (J.A. Woolam, Co., Inc). The refractive index (*n*) and extinction coefficients (*k*) of p-NiO films are also obtained from this equipment, values of which are plotted in Figure S2. Field emission scanning electron microscopy images are taken using FE-SEM JEOL JSM-7001F at 5 kV electron accelerating voltage. Meanwhile, optical spectra measurements are collected using an optical set-up consisting of integrated microscope and spectrometer system. In a typical measurement, the entire sample is illuminated with an unpolarized light source (tungsten lamp) and the reflected light was collected by a 20× objective lens of the microscope system. The data are then processed by the integrated spectrometer (Andor Kymera 328i) equipped with a CCD camera (DV401A-BVF) detector. In all the measurements, a silver mirror is used as reference for the reflection signal. To ensure reasonable signal intensity, the slit area is set at 50 × 50 μm^2^, and the reflection spectra of the samples are taken at the wavelength range of 500–900 nm. Since the transmission is essentially nil, the absorption is taken as *A* = 1 – *R*.

## 3 Results and discussion

### 3.1 Periodic square lattice FDTD simulations

**Figure 1:**
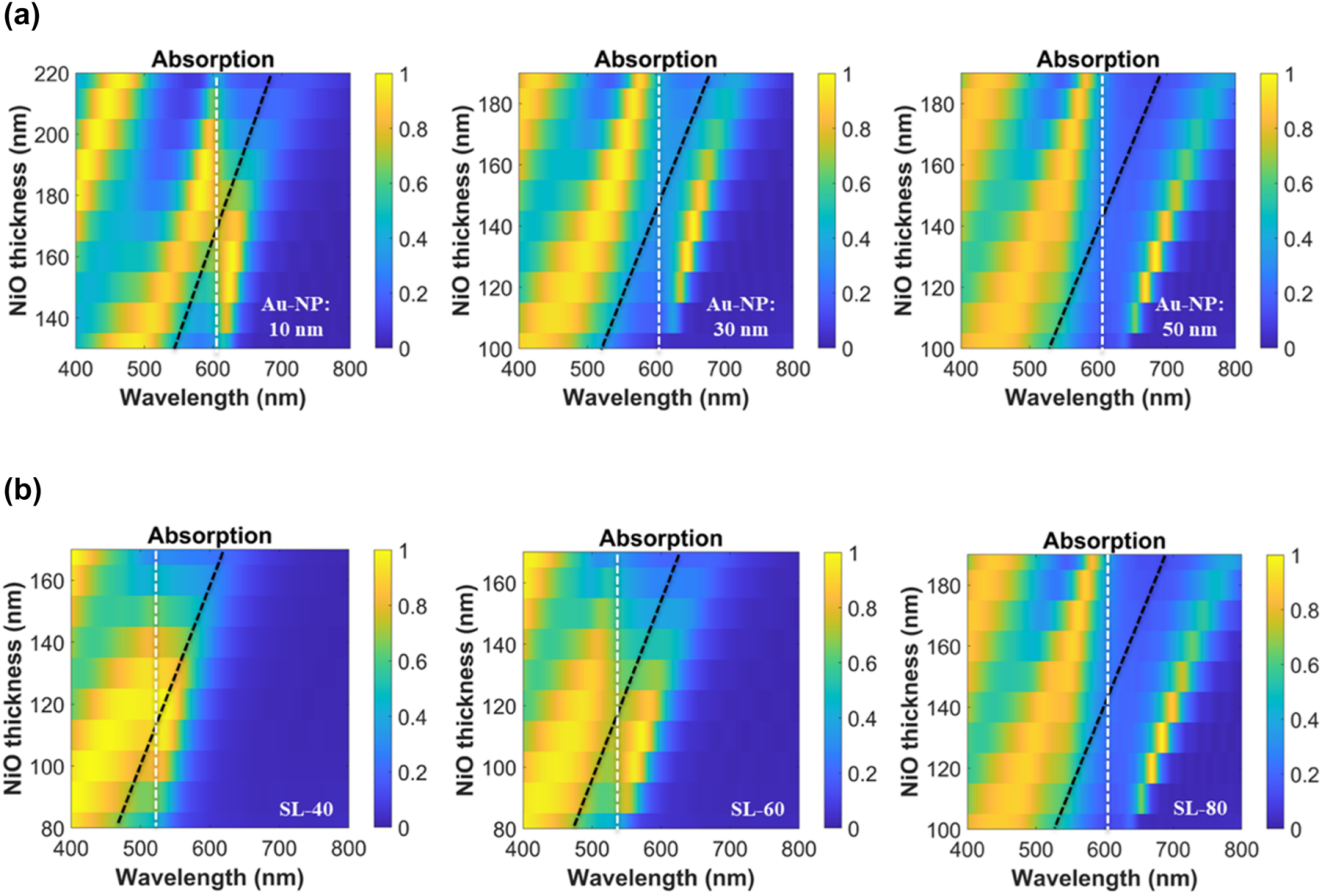
Absorption mapping of SL-ANA nanostructures exhibiting the hybrid polariton states upon strong coupling of LSPR and Fabry–Pérot mode. (a) Nanoparticle size effect at SL-80. (b) PSC effect at constant Au-NP size of 50 nm.

In this study, the Au-NPs size is varied at 10 nm, 30 nm, and 50 nm to investigate the influence of NPs size in the coupling strength between LSPR and Fabry–Pérot mode. Figure 1(a) depicts the absorption mapping of 10 nm, 30 nm, and 50 nm Au-NPs at a constant PSC of 80 % (SL-80). The anticrossing behavior is clearly exhibited across all the Au-NPs size as evident from the existence of the UPB and LPB upon coupling. Furthermore, it is apparent that the larger the NP size induces higher splitting energy between the hybrid states. The bare plasmonic mode and bare cavity mode for the individual cases of isolated plasmonic structures and dielectric cavities are presented in the Supporting Information (Figure S3).

**Figure 2:**
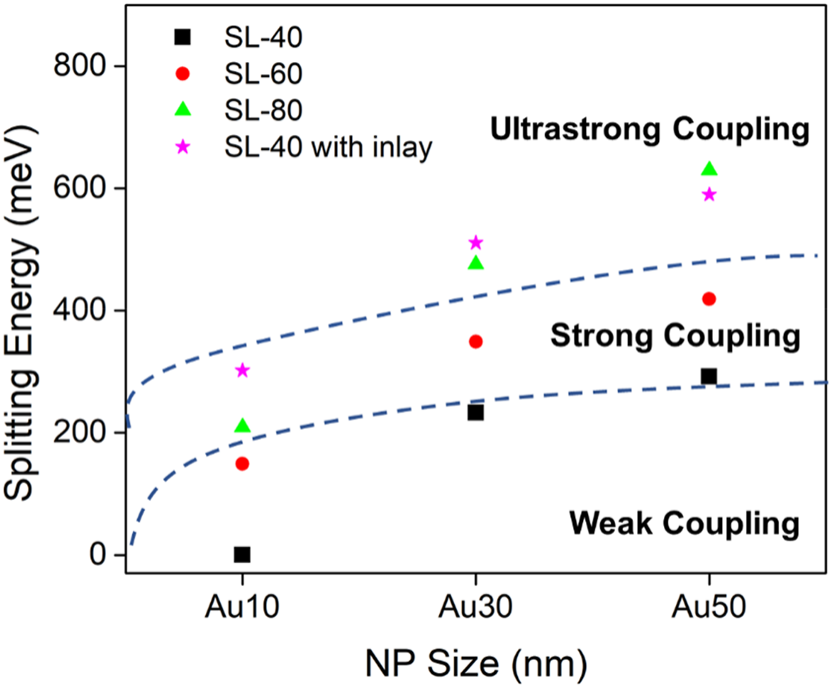
Coupling regimes between LSPR and Fabry–Pérot mode in the SL-ANA nanostructures at varying NPs size and PSC (SL-40, SL-60, and SL-80).

The effect of the PSC of nanoparticles is also probed using square lattice (SL) unit cells and is varied at 40 %, 60 %, and 80 %, denoted as SL-40, SL-60, and SL-80, respectively. This set-up represents a periodic array of nanoparticles with varying particle density and can potentially be fabricated using template-assisted self-assembly of metallic nanoparticles reported in literature [28], [29], [30]. The maximum PSC is set at 80 % where the interparticle distance is zero, that is, square close-packed arrangement. The minimum PSC is limited at 40 % where the Rabi splitting energy of the structure (292 meV) with Au-NPs of 50 nm is already very close to the average losses of the coupled system (266 meV). The values of the coupling parameters derived from the spectral fittings of the SL systems are given in Supplementary Information (see Table S1) while a summary of the corresponding coupling regimes is shown in Figure 2.

The numerical simulations reveal that at the same particle size, the higher PSC resulted to larger splitting energy as depicted in Figures 1(b) and 2. Consequently, as the PSC decreases, only the largest nanoparticle size is able to sustain the strong coupling regime condition. For the structure with Au-NPs diameter of 30 nm, although the coupling between LSPR and cavity mode at SL-40 shows a splitting of the absorption band, the effective dissipation of the hybrid states (258 meV) exceeds the coherent energy exchange (233 meV) between the two modes and, thus, the strong coupling condition 2*g* > [*γ*_UPB_ + *γ*_LPB_]/2, cannot be attained. In the 10 nm Au-NP, the strong coupling transitions to weak coupling regime at PSC of 60 %. A further decrease of PSC to 40 % results to a single resonance, in contrast to the anticrossing behavior associated with strongly coupled system. Thus, for the nanoparticle size range of 10 nm–50 nm, the larger nanoparticle has the greater possibility of reaching the strong coupling regime at a relatively lower PSC when compared to the smaller nanoparticle.

**Figure 3:**
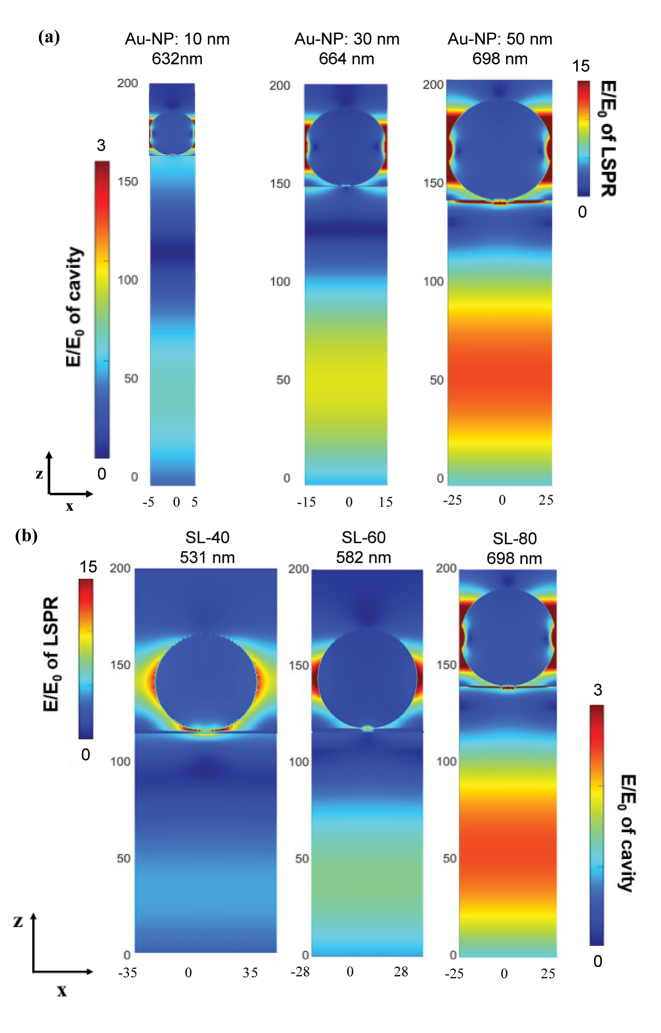
Electric field profile around AuNP/p-NiO at resonant wavelength (LPB) of SL-ANA nanostructures. The position *z* = 0 corresponds to the interface of p-NiO and Au film. The larger NPs size and higher PSC lead to greater field confinement in the Fabry–Pérot mode. (a) Nanoparticle size effect at SL-80. (b) PSC effect at Au-NPs size of 50 nm.

To elucidate the physical origin of the larger splitting energy associated with higher PSC and larger NPs size, electrodynamics simulations are performed by FDTD method. It can be noted from the electric field profile of the nanostructures at their resonance wavelengths as shown in Figure 3(a), that at the same PSC, the nanostructure with larger NPs size exhibits greater electric field enhancement inside the Fabry–Pérot mode. Likewise, at a constant nanoparticle size, the system with higher PSC reveals a greater field confinement inside the Fabry–Pérot mode. Consistently, the largest splitting energy is always associated with the strongest field enhancement in the Fabry–Pérot mode, which is attained with the larger NPs size (50 nm) and highest PSC (SL-80) as depicted in Figure 3(b). This suggests that the coherent energy exchange between the strongly coupled modes formed a channel for energy confinement in contrast to the tendency of weakly coupled resonators to undergo radiative decay into free space. This is relevant in solar energy conversion where it has been reported that the high optical mode intensity along the cavity mode is closely related to the enhancement in quantum yield under the strong coupling regime [23]. In addition, the stronger electric field resulting from strong coupling has been utilized to achieve high sensitivity in sensing applications. In the Supporting Information (Figure S4), we present simulations of refractive index sensing, providing evidence that higher sensitivity can be observed under a strongly coupled regime compared to a weaker coupling condition.

The influence of NPs size and PSC on the strength of coupling between the LSPR and Fabry–Pérot mode is in agreement with the behavior of a coupled system where the strength of light–matter interaction, *g*, is given by:

$$g=\frac{\hbar \Omega }{2}=\mu \cdot E_{0}$$

where *g* is the coupling strength, *ℏ*Ω is the Rabi splitting energy, *μ* is the dipole moment of the plasmon mode, and *E*_0_ is the electric field confined in the cavity [6], [7]. Dipole moment in plasmonic modes refers to the distribution of free current density within a plasmonic nanostructure. The multipole decomposition of Au NPs for both the upper and lower branches are shown in the Supporting Information (Figure S5), highlighting the significant role played by the electric dipole. Since the larger NP size increases the density of dipoles in the nanoparticles [31], the 50 nm Au-NPs have relatively stronger coupling interaction compared to the 10 nm and 30 nm Au-NPs at the same PSC. Moreover, as revealed from electrodynamics simulations, higher electric field confinement is achieved with larger nanoparticle size and higher PSC. As a result, the ANA structure with 50 nm Au-NPs can satisfy the strong coupling condition even at SL-40.

It is now demonstrated that both the NPs size and its PSC on top of the Fabry–Pérot mode significantly affects the attainment of the strong coupling regime for the studied systems. At SL-40, both the heterostructures with Au-NPs of 10 nm and 30 nm cannot fulfill the requirement for strong coupling. It is, however, reported in literature that by inlaying the metallic nanoparticle partially into the Fabry–Pérot mode, the strength of coupling can be enhanced [23], [26]. Therefore, to verify whether the simulation method in this study is in reasonable agreement with recent reports, the SL-40 systems are considered, and their arrangement is modified such that the nanoparticles are partially inlaid into the Fabry–Pérot mode by 50 % of their size, which means the inlaid depth is equal to the radius of the NPs. Indeed, FDTD results confirmed that this arrangement effectively increases the interaction between the two modes and the resulting Rabi splitting energies exceed the average losses in the coupled systems as listed in Table S1. To further understand the physical origin of this enhanced modal coupling, the electric field profiles of the SL-40 nanostructures before and after incorporating partial inlay of the Au-NPs are obtained from the electrodynamic simulations. Similar to the NPs size and PSC effect observed earlier, it is evident from the electric field profiles that the heterostructures in which the NPs are partially embedded into the dielectric layer allowed for a greater field confinement in the Fabry–Pérot mode (see Figure S6 in the Supporting Information). As mentioned earlier, the strength of coupling interaction is directly proportional to the electric field confined inside the cavity mode. Also, partial inlaying of the NPs enables spatial overlap between the two modes and thus results a more enhanced coupling.

### 3.2 Hexagonal lattice close-packed FDTD simulations

Since the simulation results suggest that the coupling interaction between LSPR and Fabry–Pérot mode in the proposed nanostructure benefits from higher PSC, we then explore ANA systems wherein the NPs are arranged in the HL-CP configuration in order to achieve even higher PSC than SL-80 system. FDTD calculations are conducted for ANA systems in HL-CP configurations with varying IPD across the particle size range considered in this study to provide a more comprehensive outlook on the onset of strong coupling for this type of nanostructures. The transition from strong coupling regime to the weak coupling regime is observable from the calculated absorption spectra as consolidated in Figure S7(a)–(c) (see the Supporting Information) for Au-NPs size of 10 nm, 30 nm, and 50 nm, respectively. Similar to previous cases, Lorentz fittings are utilized to quantify the Rabi splitting energy and effective dephasing of the hybrid states and thereby assess if the structures fulfill the strong coupling regime condition.

**Figure 4:**
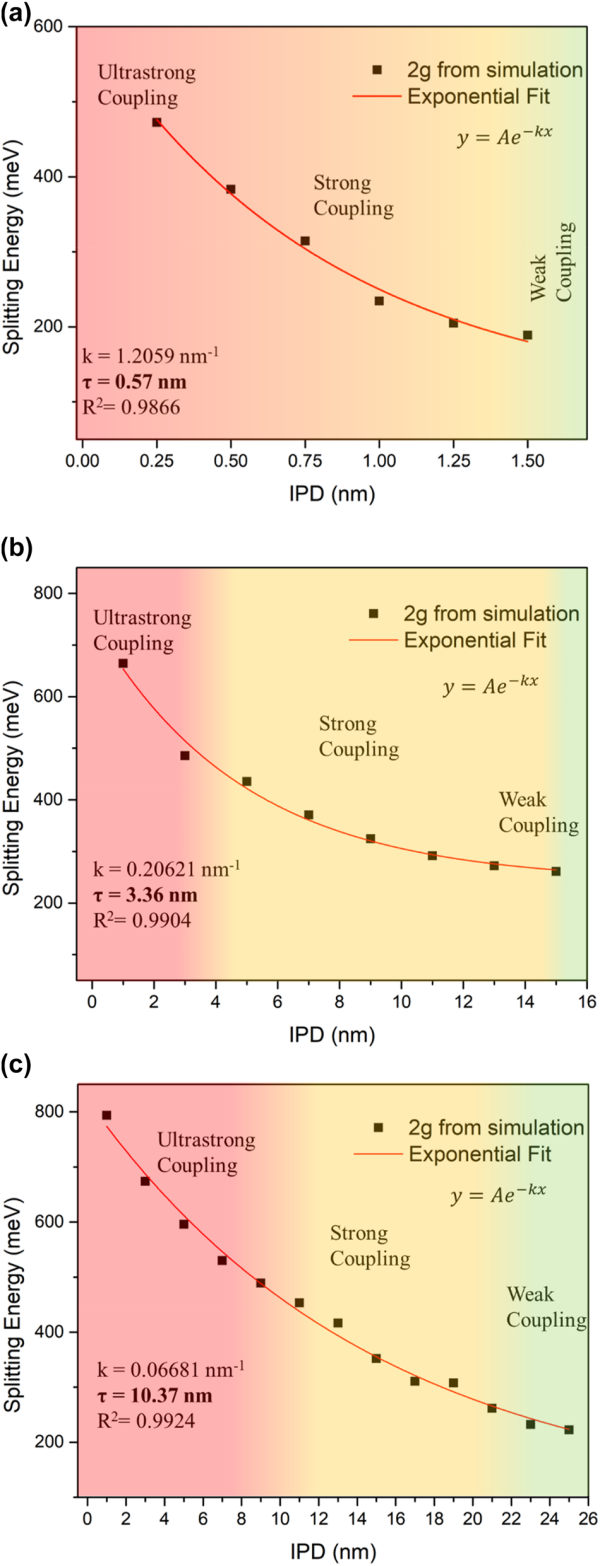
IPD effect. The Rabi splitting energies of the ANA systems at varying IPD can be fitted with a first-order exponential function. The derived parameter *τ* corresponds to a critical length where the coupling regime transitions from ultrastrong to strong coupling regime. (a) Au-NPs: 10 nm, (b) Au-NPs: 30 nm, (c) Au-NPs: 50 nm.

Consistent with the trend observed in the periodic SL arrangement, the Rabi splitting energy increases as the interparticle distance decreases (analogous to larger PSC). Similarly, the larger NP size can sustain the strong coupling regime over a larger expanse of interparticle distance. The strong coupling condition is maintained up to a IPD to NP diameter ratio of 0.13 for 10 nm Au-NPs, 0.36 for 30 nm Au-NPs, and 0.40 for 50 nm Au-NPs. This is similar to the SL arrangement where the strong coupling can be realized at a relatively lower PSC by using larger nanoparticle size. The plot of IPD dependence of the splitting energies for different NPs sizes are illustrated in Figure 4. It is apparent from the trend that the Rabi splitting energy decays exponentially with respect to increasing IPD. The data fit very well with a first order exponential equation, which can be expressed as:

$$y=Ae^{-k\left(x\right)}$$

where *y* represents the Rabi splitting energy, and *x* is the IPD. The derived fitting parameters are *k* and *τ* that represent the decay rate and ln(2)/*k*, respectively. Intriguingly, it is discovered that the value of *τ* coincides to the critical length where the coupling strength transitions from ultrastrong coupling regime (2*g* > 0.2*ℏω*_0_) to strong coupling regime (*γ*_average_ < 2*g* < 0.2*ℏω*_0_). Such insights are highly beneficial since the self-assembly of metallic nanoparticles into ordered arrays has been continuously improved in the recent years and can be utilized to fabricate such types of nanostructures.

Another noteworthy feature of the HL-CP nanostructures is the emergence of an additional polariton branch when the NPs size is 50 nm and the IPD approaches the touching limit (see Supplementary Note 5). Ordered arrays of intermediate sized metallic nanoparticle give rise to multipolar modes and their plasmonic coupling results to splitting of the plasmonic band [32]. Generally, the duals bands are attributed to the red-shifted dipolar mode and the emergence of blue-shifted higher order modes especially the quadrupole mode [33]. We speculate that the plasmonic coupling among the NPs and its further coupling to the Fabry–Pérot mode gave rise to the final hybridization, which results in the formation of three hybrid states. It is noted that the additional resonance in the ANA HL-CP system vanishes when the IPD exceeds 0.75 nm (see Figure S8(a)), consistent with several studies, which remark that the appearance of higher order modes in strongly coupled plasmons is extremely sensitive to the distance between nanoparticles [30], [34]. To further verify the nature of the hybrid states, we conducted multipole decomposition analysis for the three polariton branches. The results, shown in Figure S8(b), reveal that higher order modes played a role in the modal coupling between plasmonic mode and nanophotonic mode when the nanoparticles size is large enough ( ≥ 50 nm) and there is diminutive interparticle distance approaching the touching limit. In particular, it is noted that both the upper and middle polariton branches of ANA structure with 50 nm Au-NPs are primarily contributed by the electric dipole mode and higher order electric quadrupole mode while the lower energy branch is mostly dominated by the dipole mode.

### 3.3 Experimental realization of ultrastrong coupling regime

**Figure 5:**
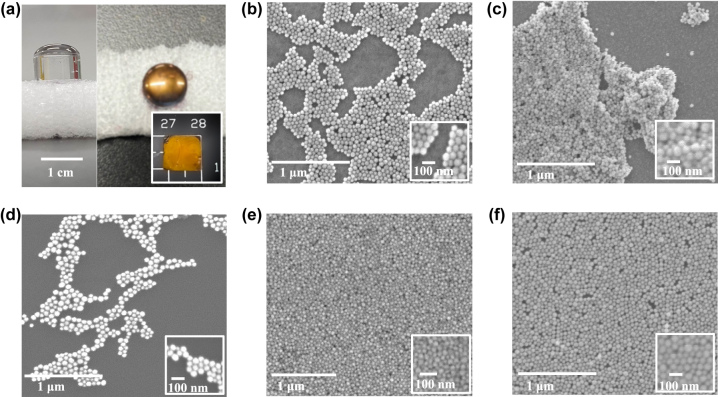
Experimental realization of ultrastrong coupling. (a) Air–liquid interface self-assembly of Au-NPs on 1-cm quarts vial was filled with the subphase deionized water. Au-NPs in chloroform was spread dropwise onto the water surface and a gold sheen was evident upon the self-assembly of NPs. (b) Short-range order of Au-NPs monolayer due to insufficient washing of NPs (2×) after phase transfer. (c) Aggregation of Au-NPs upon excess washing of NPs (6×) after phase transfer. (d) Network of chain-like structure of Au-NPs at low particle concentration. (e) Long-range order of close-packed Au-NPs monolayer (ANA-30) upon optimization of particle concentration and washing time after phase transfer. (f) Long-range order of close-packed Au-NPs monolayer (ANA-50) upon optimization of particle concentration and washing time after phase transfer.

ANA structure nanodevices are fabricated as a proof of concept for the theoretical results. To maximize the Rabi splitting energy as well as confirm the NPs size effect on the coupling strength, Au-NPs with sizes 30 nm and 50 nm are chosen, designated as ANA-30 and ANA-50, respectively. The Au-NPs are deposited on top of the p-NiO/Au film by utilizing a facile air/liquid interface self-assembly described in the methods section. Optical images of the self-assembly process are shown in Figure 5(a). Several factors are found to be crucial in achieving the target close-packed monolayer of Au-NPs loaded on top of the dielectric film. First, the transfer of Au-NPs from aqueous to organic solvent is very sensitive to the amount of alkanethiol ligand (1-dodecanethiol, DDT) used to facilitate the phase transfer. Generally, an excess amount of DDT is suggested to ensure complete coverage of the NPs and efficient transfer across the phases. Moreover, the addition of prestabilizer poly(ethyleneglycol) methyl ether thiol (PEG-SH) before the NPs solvent exchange is proven to be essential to prevent agglomeration of Au-NPs during the phase transfer. Second, upon the successful transfer of the nanoparticles in the organic solvent, the excess ligands must be washed away. Insufficient washing of the excess DDT resulted in a monolayer film lacking long range order as depicted in Figure 5(b) while excess washing led to aggregation of the NPs as shown in Figure 5(c). Lastly, the concentration of the final Au-NPs dispersion to be spread on top of the water surface must be tuned to ensure large surface coverage and long range close-packing of NPs. It is possible to occupy the entire water surface with a uniformly well-dispersed yet nonclose-packed Au-NP monolayer as observed in Figure 5(d). The concentration of the dispersion is varied until a reasonably close-packed monolayer of Au-NPs is obtained as shown in Figure 5(e) for ANA-30 and Figure 5(f) for ANA-50. It should be noted that defects such as vacancies are also present as such are among the challenges yet to be fully addressed from self-assembly of 2D monolayer of spherical Au-NPs. The Au-NPs are transferred to the substrate by gently touching the p-NiO on top of the water surface where the close-packed monolayer is self-assembled.

**Figure 6:**
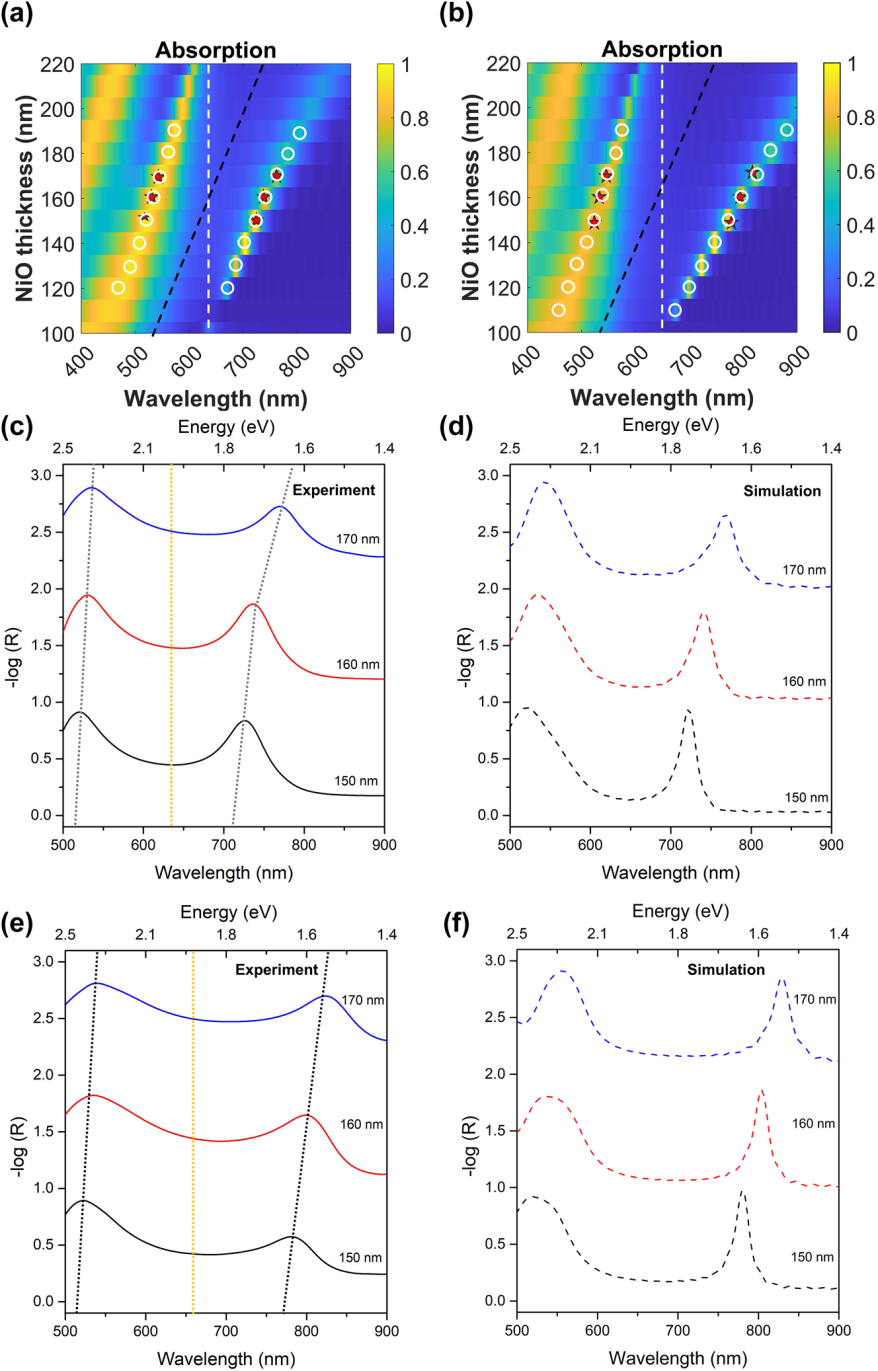
Dispersion behavior of the coupled system. (a) Absorption mapping of ANA-30. (b) Absorption mapping of ANA-50. The white circle and red star indicate the resonance position of the FDTD and experimental hybrid states, respectively. (c) Experimental absorption spectra of ANA-30 systems at different NiO thickness. The yellow dotted line represents the LSPR energy, and the black dotted curve traced the dispersion of the hybrid polaritonic states. (d) FDTD calculated absorption spectra of ANA-30 systems. (e) Experimental absorption spectra of ANA-50 systems at different NiO thickness. The yellow dotted line represents the LSPR energy, and the black dotted curve traced the dispersion of the hybrid polaritonic states. (f) FDTD calculated absorption spectra of ANA-30 systems.

To map the dispersion behavior of the coupled system, ANA devices with various p-NiO thickness (150 nm, 160 nm, 170 nm) are fabricated. The Rabi splitting energy can then be directly evaluated from the Lorentzian fitting of the optical spectra of the coupled system as is commonly done in literature [35]. Figure 6(a) and (b) depict the FDTD calculated absorption mapping of the ANA-30 and ANA-50 systems. For comparison, the resonance positions of the UPB and LPB are superimposed as white circles for the simulation data and red star for the experimental data. Overall, the experimental results are in reasonable agreement with the FDTD simulation. The UPB and LPB hybrid states are clearly manifested in the experimental absorption spectra as given in Figure 6(c)–(f), and their resonance energies closely resemble that of the ANA HL-CP with interparticle distance of 1 nm.

The linewidths of the bands are broadened compared to the simulation, and this could be attributed to the fact that there is an inhomogeneous particle size and interparticle distance between the Au-NPs [25]. The size distribution of Au-NPs is determined by analyzing the SEM images. The average NPs size is found to be 50.15 nm with a standard deviation of 4.25 nm (Figure S9). In addition, the vacancies and defects present in the monolayer could have also contributed to the additional damping in the hybrid states. Nevertheless, based on the results of spectral fitting, the fabricated ANA heterostructures satisfactorily achieve the strong coupling condition with their immense Rabi splitting energy of 655 meV (ANA-30) and 770 meV (ANA-50), surpassing their average dephasing of 269 meV and 351 meV, respectively. More importantly, the coupling of LSPR and Fabry–Pérot mode of the fabricated devices corresponds to a remarkable splitting energy to bare (plasmon) energy ratio (2*g*/*ω*_0_) of 0.35 (ANA-30) and 0.4 (ANA-50), indicating the fabricated devices reached the ultrastrong coupling regime.

## 4 Conclusions

In summary, a systematic study of the parameters contributing to the realization of modal strong coupling between the LSPR mode of plasmonic Au-NPs and Fabry–Pérot mode (p-NiO/Au film) is conducted using FDTD simulations. By tuning the size, PSC, IPD, and arrangement of nanoparticles on top the Fabry–Pérot mode, the different coupling regimes (weak, strong, ultrastrong) can be explored. The numerical simulations reveal that at a constant PSC of NPs, the larger NP size (50 nm) results to stronger coupling as evident by the larger Rabi splitting energy. Meanwhile, at a constant NP size, higher PSC (i.e., smaller IPD) allows stronger interaction between the modes. These trends can be attributed to the higher density of dipoles in the larger NPs, and the greater field confinement in the cavity mode as the NPs size and PSC is increased. The partial inlay of NPs into the Fabry–Pérot mode also enhances the coupling since it allows spatial overlap between the two modes. Interestingly, the possibility of a third hybrid state is hinted for closely packed Au-NPs (size ≥ 50 nm) with diminutive interparticle distance approaching the touching limit. Multipole decomposition analysis suggests that the higher order modes contribute to the emergence of this additional polariton branch.

Going beyond normal strong coupling, the larger nanoparticle size, close packing (also smaller IPD), and partial inlay of NPs into the Fabry–Pérot mode are the factors identified toward realizing ultrastrong coupling regime in the studied nanostructures. In accordance with the theoretical findings, nanodevices consisting of 30 nm and 50 nm Au-NPs/p-NiO/Au film (ANA-30 and ANA-50) are fabricated. With its low cost and potential scalability, the facile self-assembly process demonstrated in this work provides a practical approach to explore the system. The final nanodevices exhibit giant Rabi splitting energies of 655 meV (ANA-30) and 770 meV (ANA-50), which represent a sizable fraction of the uncoupled plasmon energy, and thus satisfactorily reached the ultrastrong coupling regime. Overall, this study provides comprehensive physical insights obtained from the numerical calculations, contributing new perspectives toward the realization of higher coupling strength between LSPR and Fabry–Pérot mode in MDM nanostructures for the promising enhancement in the performance of plasmonic-based nanodevices.

## Supplementary Material

Supplementary Material Details
