# Supplementary material for: Realization of ultrastrong coupling between LSPR and Fabry–Pérot mode via self-assembly of Au-NPs on p-NiO/Au film
Source: Nanophotonics. 2024 Jan 19;13(14):2501–12. doi: 10.1515/nanoph-2023-0763 (PMC11636521; doi:10.1515/nanoph-2023-0763)
Supplement: Supplementary file 1 — Supplementary Material Details [file j_nanoph-2023-0763_suppl_001.docx]

Realization of Ultrastrong Coupling between LSPR and Fabry-Pérot Mode via Self-assembly of Au-NPs on p-NiO/Au film

*Alexis Angelo R. Garcia,*^1^ *Cheng-An Mao,*^1^ *Wen-Hui (Sophia) Cheng*^1,2,^*

^1^Department of Materials Science and Engineering, National Cheng Kung University, Tainan, Taiwan

^2^Hierarchical Green-Energy Materials (Hi-GEM) Research Center, National Cheng Kung University, Tainan, Taiwan

*Corresponding author: [wcheng@gs.ncku.edu.tw](mailto:wcheng@gs.ncku.edu.tw)

**Supplementary Information**

**Supplementary Note 1. FDTD simulation set-up**

The simulation model consists of square lattice (SL) unit cell to represent periodic array of nanoparticles with varying projected surface coverage area (PSC). For simplicity, the PSC is defined as the projected area of sphere ($A=\pi r^{2}$) over the planar area of the substrate ( $P_{x}\times P_{y}$). The PSC was then varied by adjusting the simulation pitch accordingly. At the lowest PSC (SL-40), incorporation of partial inlay of NPs into the dielectric layer was also explored. Lastly, rectangular lattice unit cell with periodic boundary conditions is utilized to simulate a hexagonal close-packed arrangement of nanoparticles on top of the nanocavity. In this set-up, the interparticle distance (IPD) was varied to infer the transition between coupling regimes.


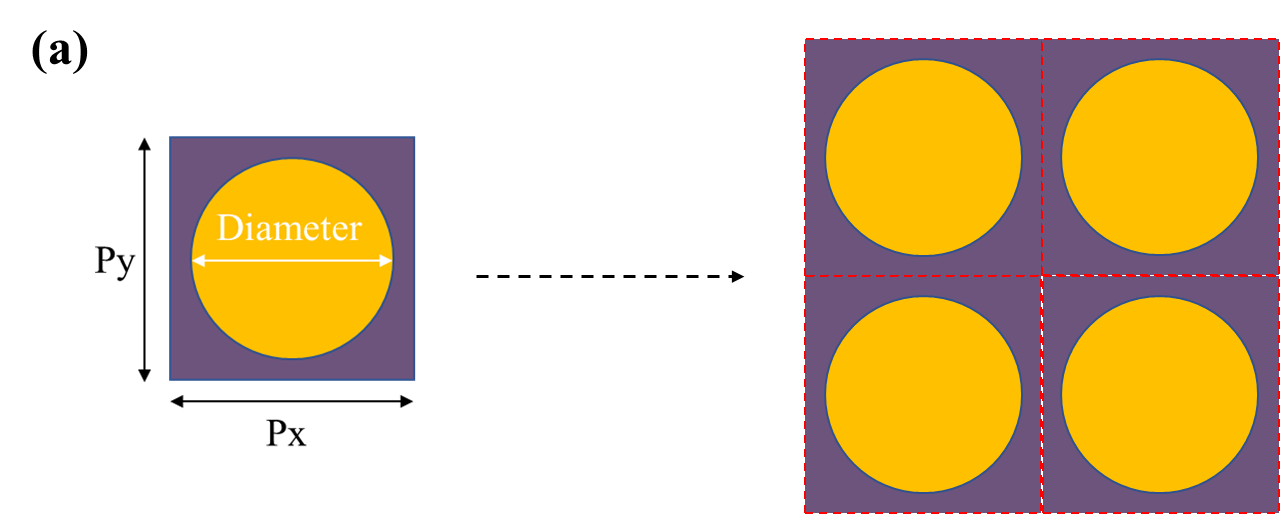


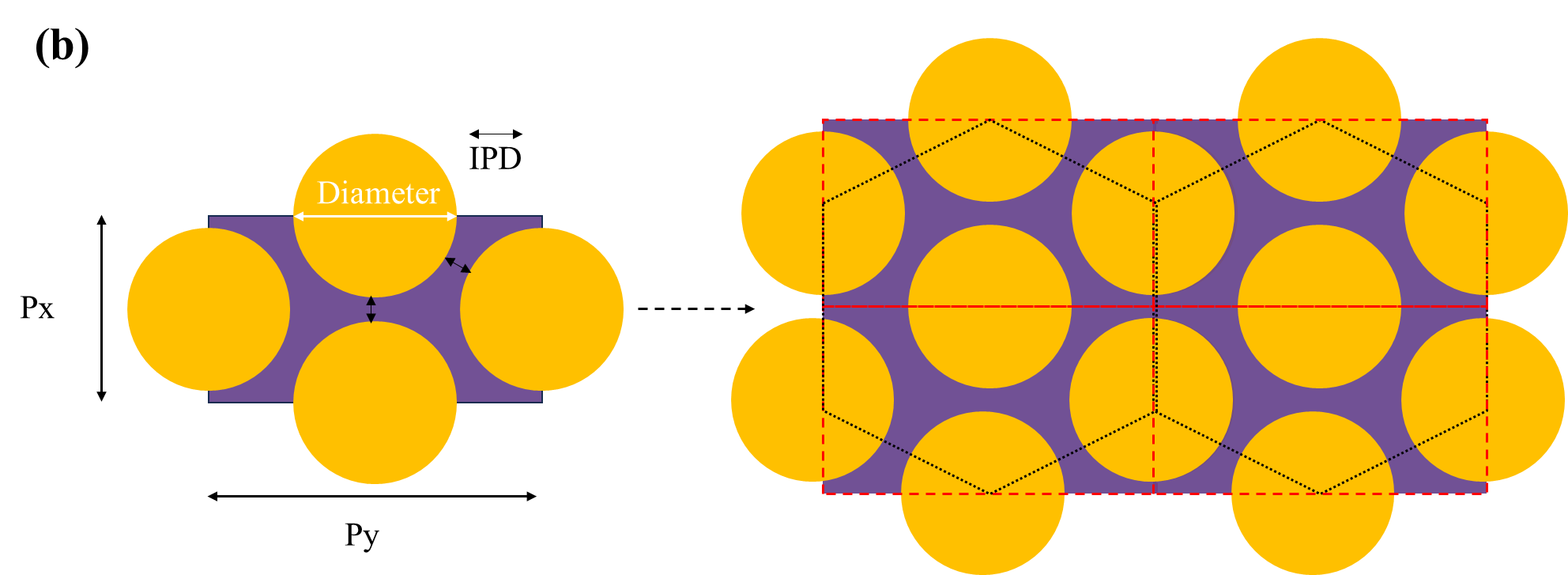


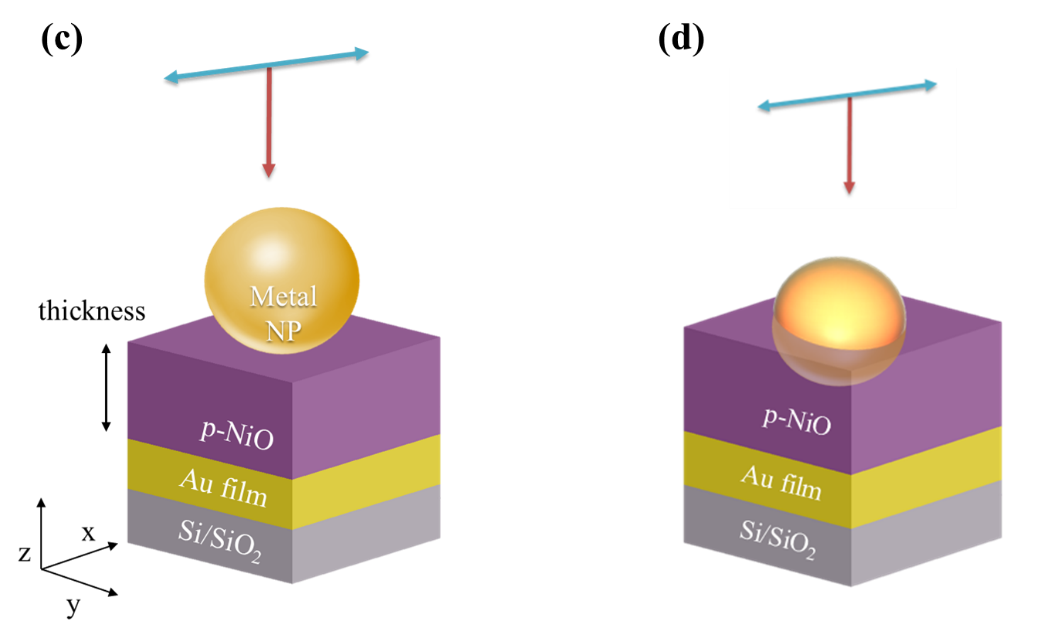


**Figure S1.** Schematic of the simulation set-up of studied systems. (a) SL unit cell representing periodic arrays of NPs with varying PSC (b) Rectangular lattice unit cell representing hexagonal close-packed arrangement of NPs, (c) SL nanostructure without inlay, (d) SL nanostructure with partial inlay.

**Supplementary Note 2. Experimental n, k values from ellipsometry**

The p-NiO layer was fabricated by magnetron sputtering and the n,k values were determined experimentally by ellipsometry. Then data was then incorporated in the FDTD simulations.


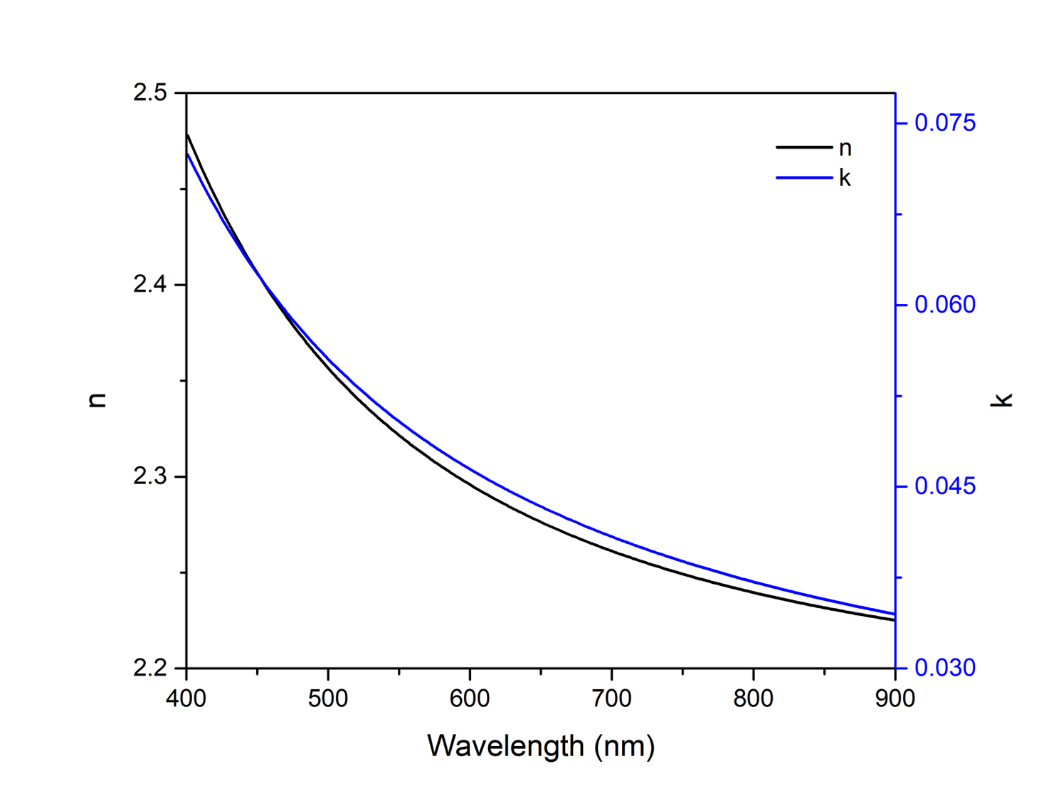


**Figure S2.** Experimental n,k values of p-NiO film


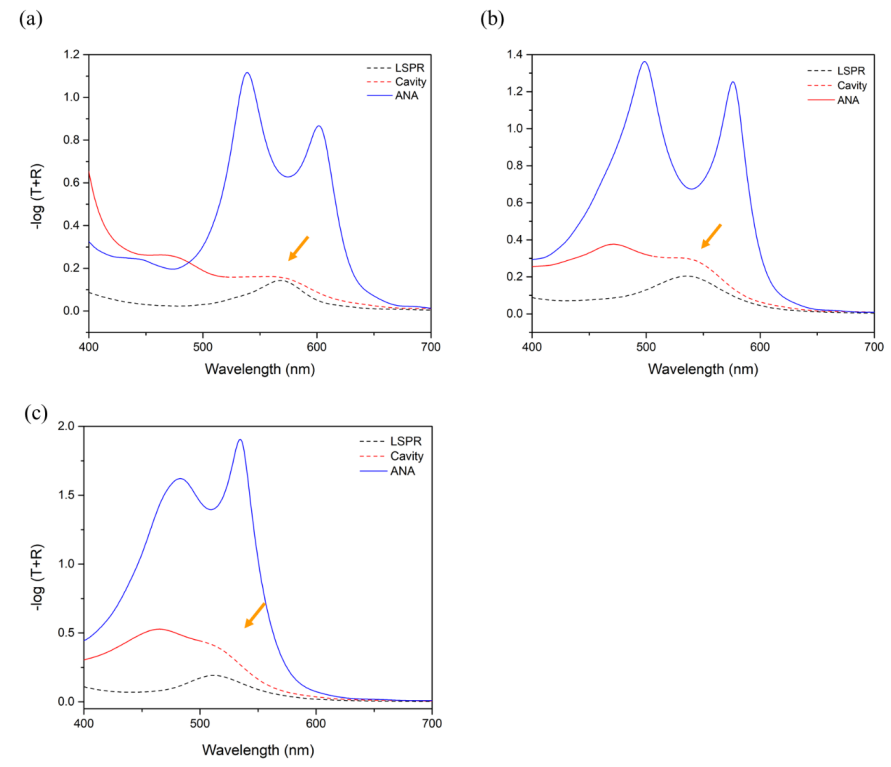


**Figure S3.** Representative absorption spectra confirming that the formation of two new hybrid states is due to the coupling of LSPR mode and cavity mode. (a) ANA SL-80, Au-NP: 10nm; (b) ANA SL-60, Au-NP: 30nm; (c) ANA SL-40, Au-NP: 50nm. The black and red dashed lines denote the individual LSPR and cavity modes, respectively, having the same resonance energy as pointed by the arrow. The solid red line indicates the higher energy cavity mode that cannot couple with the LSPR, while the solid blue line depicts the resulting band splitting upon strong coupling.

**Table S1. Coupling Parameters of SL nanostructures**

| **Particle Size** | **PSC, %** | **2g, meV** | **γ_average_, meV** | **g/ω_o_** | **Coupling Regime** |
| --- | --- | --- | --- | --- | --- |
| **Au50 nm** | SL-40 | 292 | 266 | 0.06 | Strong |
|  | SL-60 | 419 | 282 | 0.08 | Strong |
|  | SL-80 | 630 | 196 | 0.16 | Ultrastrong |
|  | SL-40 with Inlay | 590 | 133 | 0.15 | Ultrastrong |
|  |  |  |  |  |  |
| **Au30 nm** | SL-40 | 233 | 258 | - | Weak |
|  | SL-60 | 349 | 221 | 0.08 | Strong |
|  | SL-80 | 476 | 133 | 0.11 | Ultrastrong |
|  | SL-40 with Inlay | 511 | 111 | 0.12 | Ultrastrong |
|  |  |  |  |  |  |
| **Au10 nm** | SL-40 | - | - | - | Weak |
|  | SL-60 | 149 | 264 | - | Weak |
|  | SL-80 | 209 | 143 | 0.05 | Strong |
|  | SL-40 with Inlay | 302 | 154 | 0.07 | Strong |


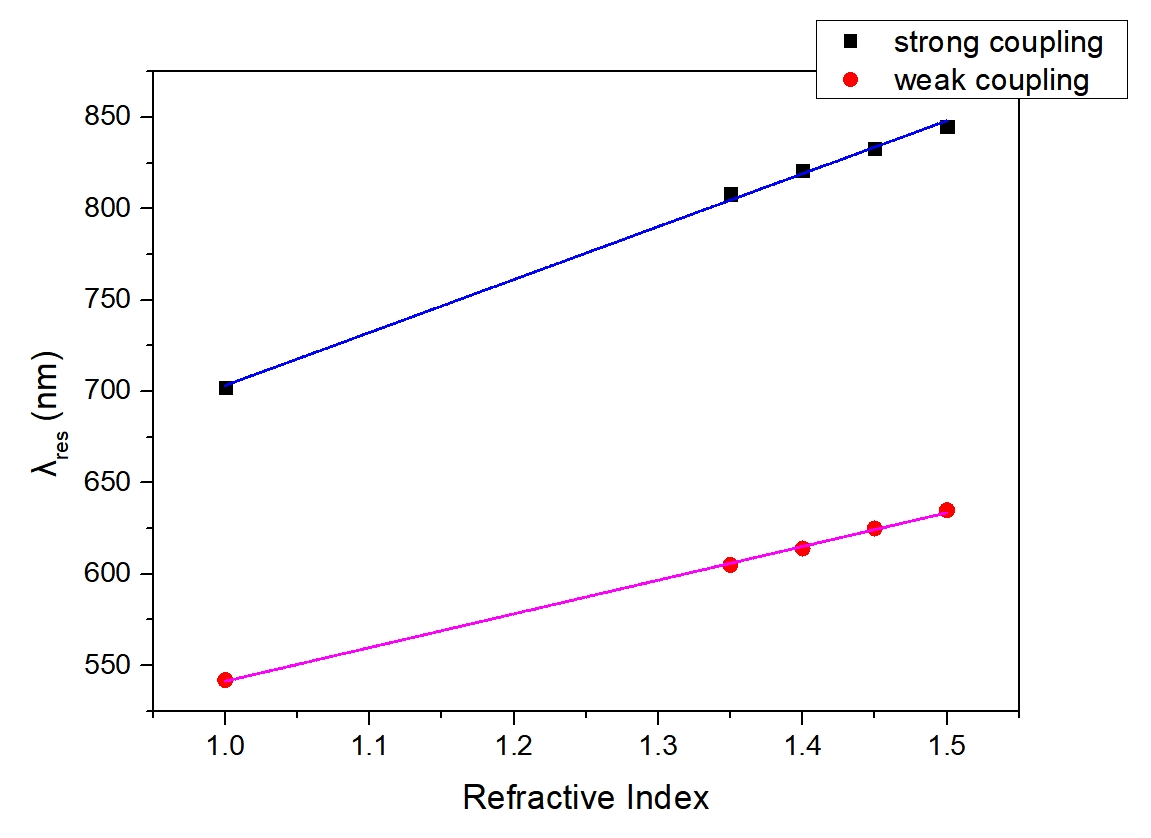


λ_res_=289.75*RI + 414.54

λ_res_=184.46*RI + 357.03

**Figure S4.** Refractive index sensing with different coupling conditions. This figure shows that the structure with strong coupling exhibits higher sensitivity, even when compared to a scenario in which plasmonic mode still couples to the cavity mode, albeit in a relatively weaker fashion.

0


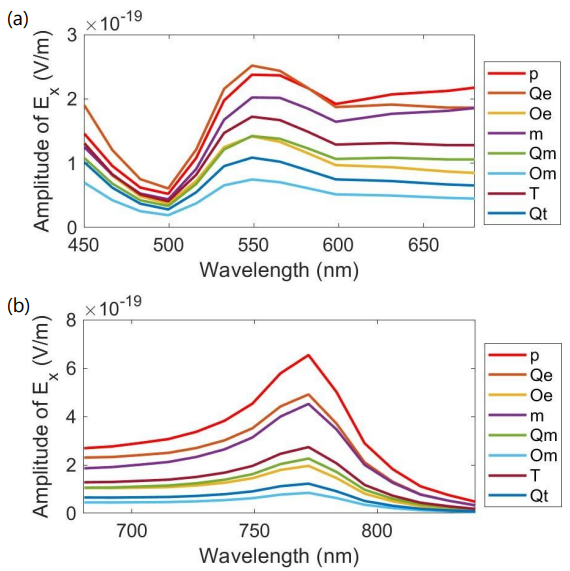


**Figure S5.** Multipole decomposition analysis of the hybrid states around Au NPs of ANA CP, Au: 30 nm structure with two resonance peaks: (a) Upper branch, (b) lower branch. These figures verify that electric dipole dominates the far-field radiation for field enhancement.

**Supplementary Note 3. Electric Field Profiles**

Electric field profiles of the nanostructures are obtained from electrodynamics simulation to gain additional insights on the physical origin of the stronger coupling (larger Rabi splitting energy) when the NP partially embedded into the dielectric layer. It was shown that the dominant factor contributing to the enhanced modal coupling is the greater field confinement in the cavity mode at such arrangements. This is consistent with the behavior of coupled system where the strength of light-matter interaction is proportional to the transition dipole moment and electric field confinement in the cavity.

**
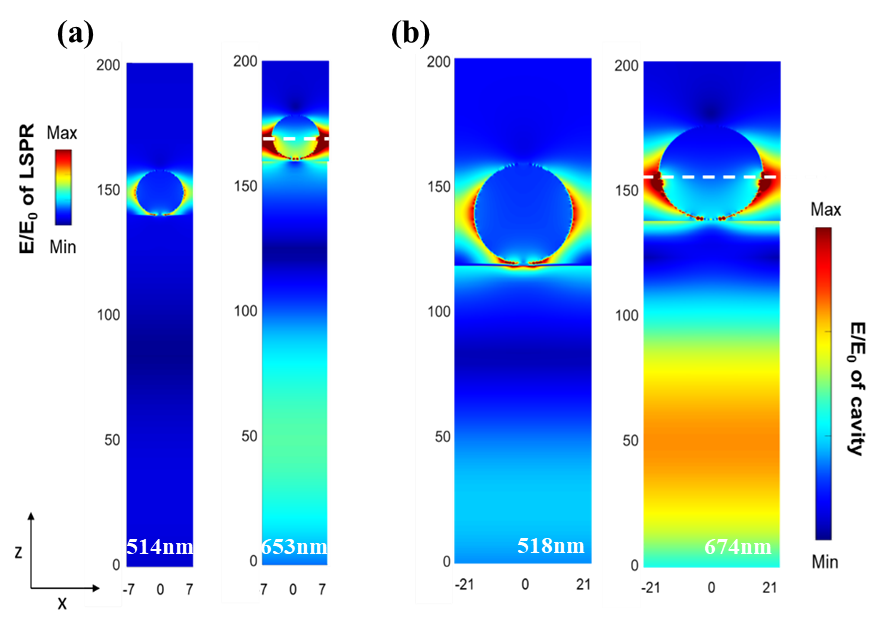
**

**
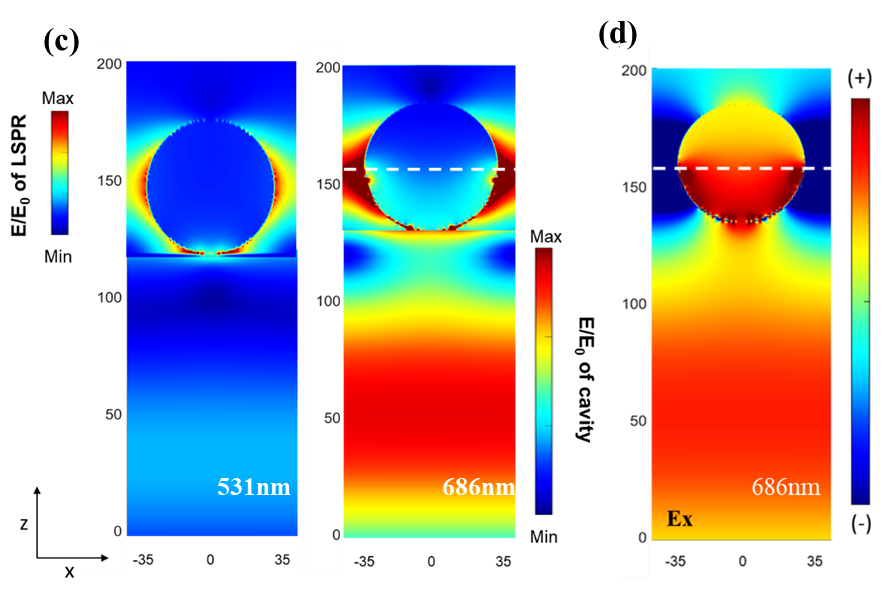
**

**Figure S6.** Electric field profile showing the effect of partially inlaying NPs into nanocavity on SL-40 nanostructures at varying nanoparticle size. (a) 10 nm, (b) 30 nm, (c) 50 nm, (d) E_x_ component of heterostructure with Au-NPs size of 50 nm showing spatial overlap of two modes. For (a) - (c): Left (no inlay), Right (partial inlay). The white dashed line marks the top on p-NiO, showing that NPs are partially inlaid into the nanocavity.

**Supplementary Note 4. Interparticle distance effect on HL-CP nanostructures.**

By varying the interparticle distances of ANA nanostructures with HL-CP configuration, the transition between weak to strong coupling can be observed.


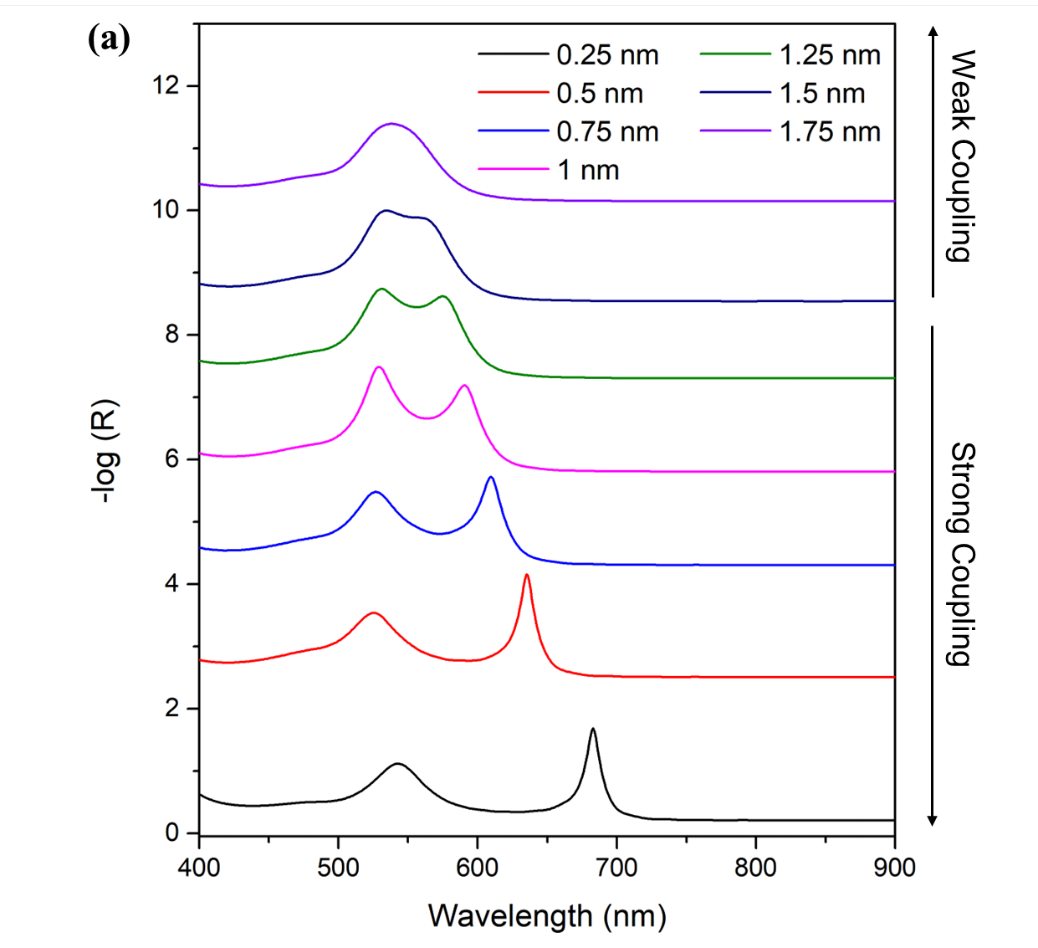


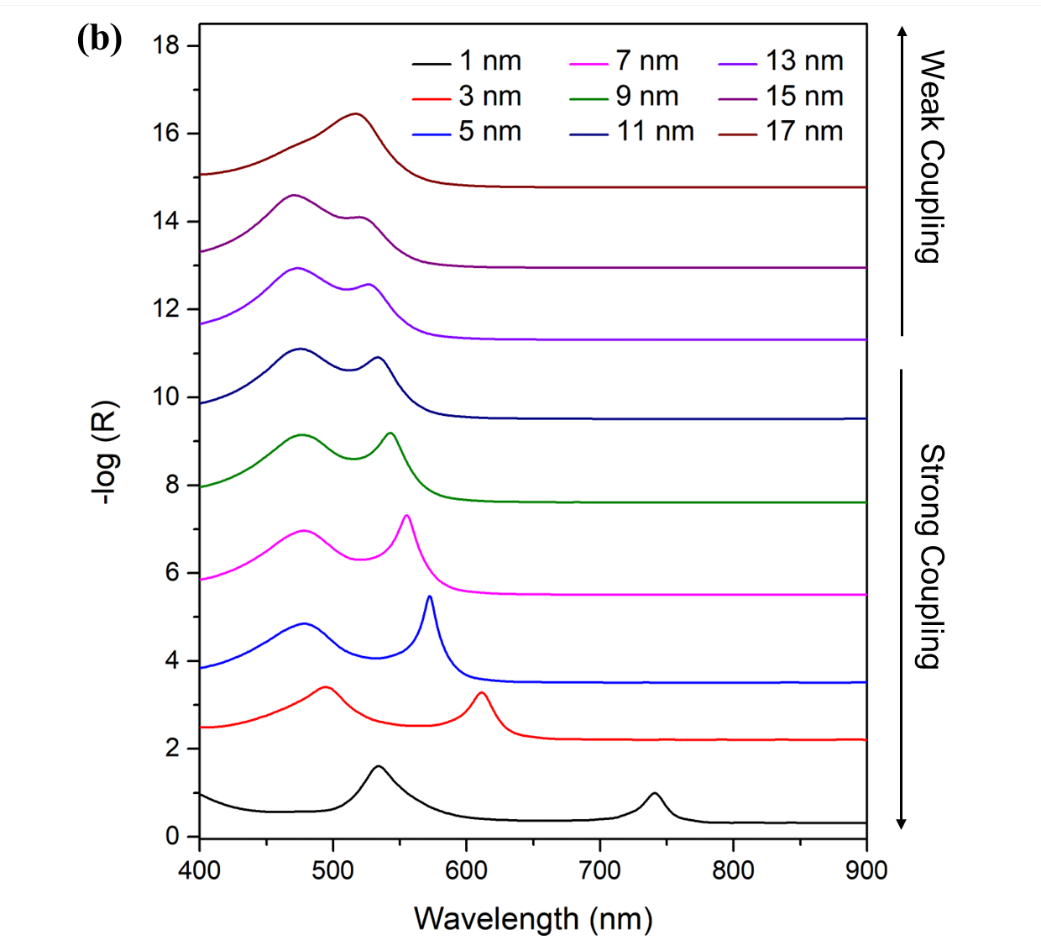


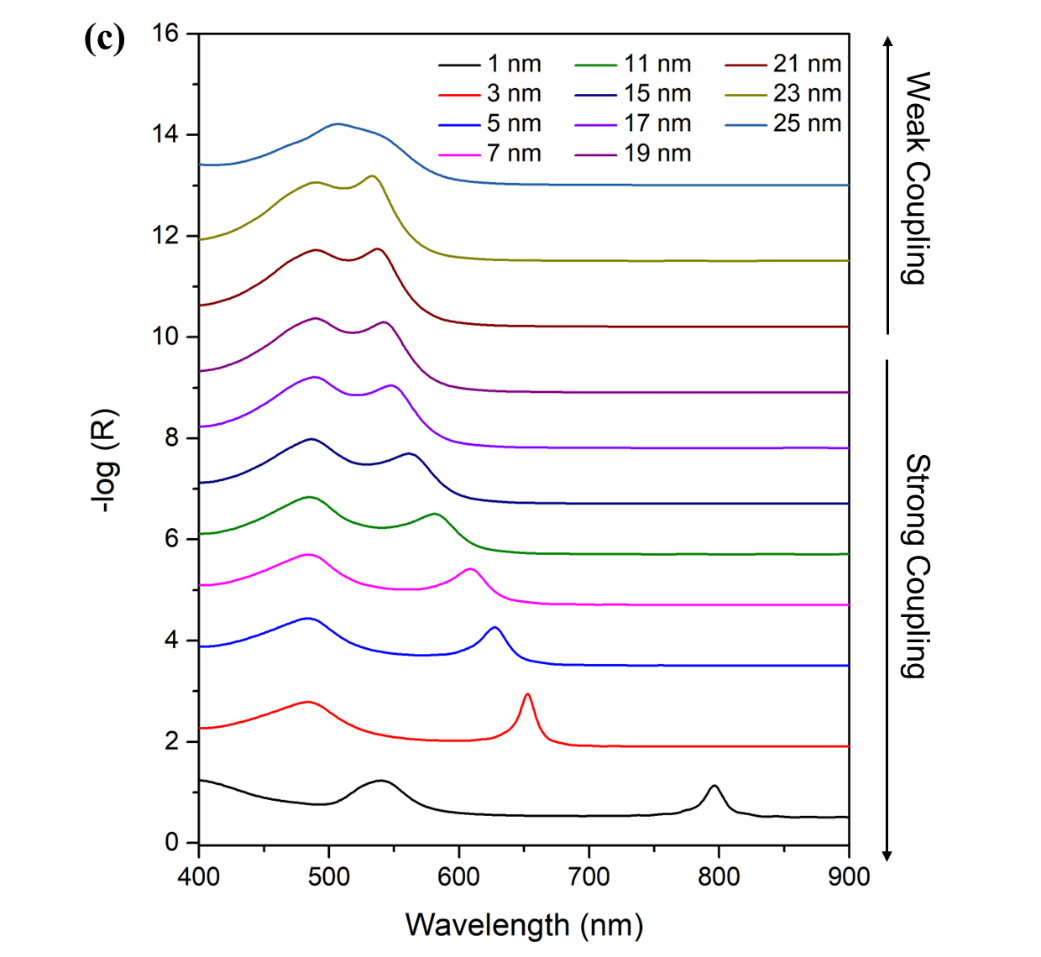


**Figure S7.** Calculated absorption spectra of ANA HL-CP nanostructures as a function of IPD at varying Au-NPs size. (a) 10 nm, (b) 30 nm, (c) 50 nm.

**Supplementary Note 5. Emergence of additional branch at HL-CP arrangement**

When the IPD approaches the touching limit for ANA HL-CP nanostructures with Au-NPs of 50 nm, the higher order plasmon mode contributes to the overall coupling and results to the formation of additional hybrid states. The multipole decomposition analysis indicates that the higher order electric quadrupole mode contributed to the upper and middle polariton branches, while the lower polariton branch is dominated by the electric dipole mode. When a gap was introduced in between the nanoparticles in the HL-CP arrangement, the disappearance of the third resonance was observed at interparticle distance of 0.75 nm.


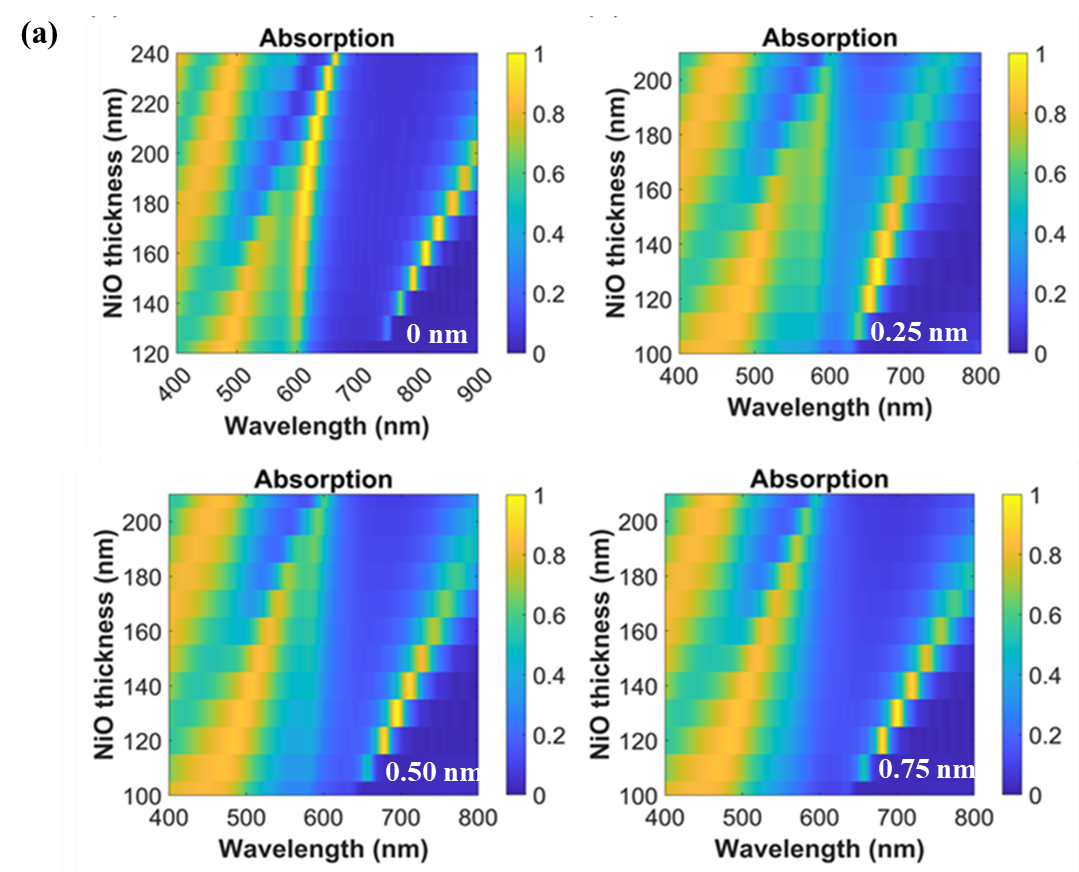


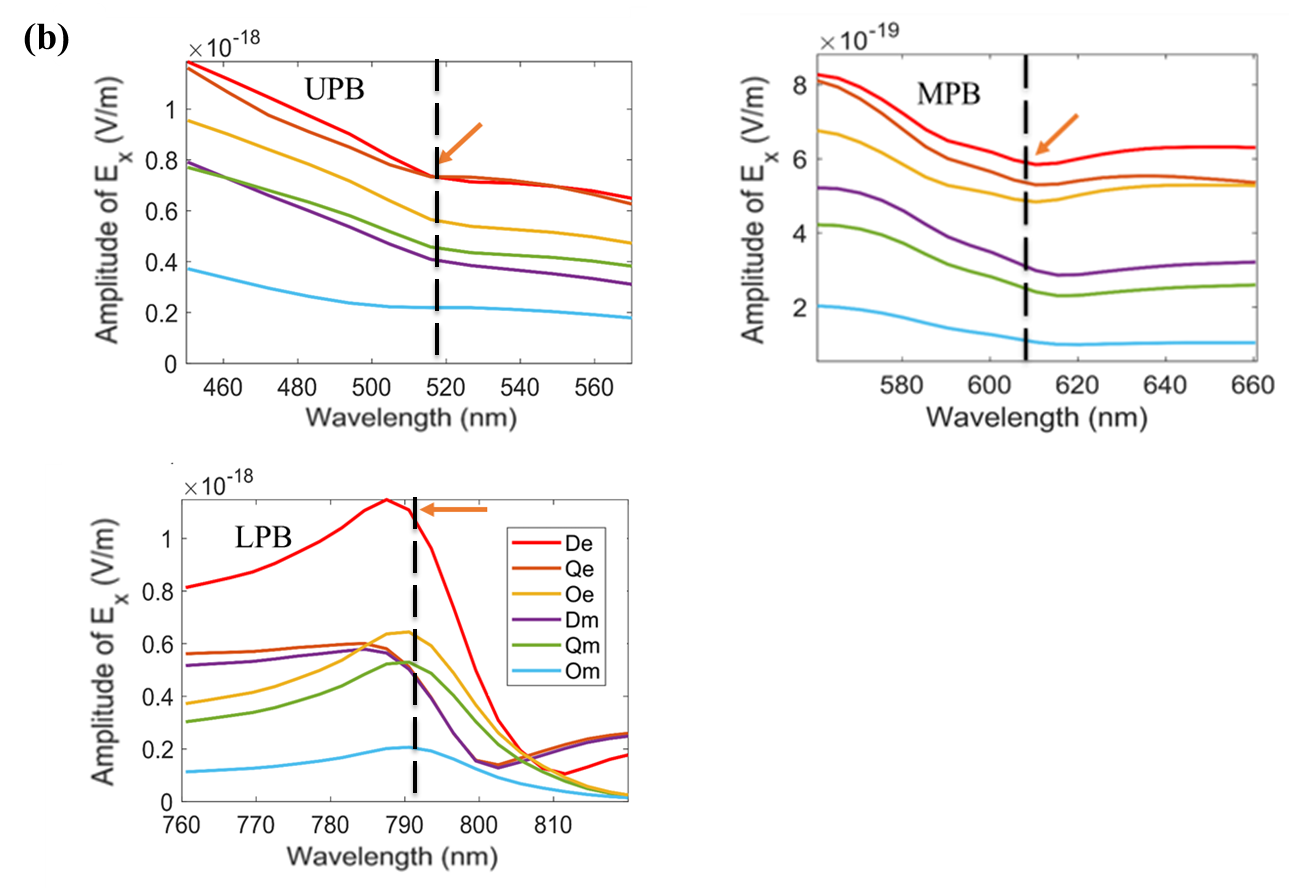


**Figure S8.** Emergence of 3^rd^ hybrid state in ANA HL-CP nanostructure with Au-NPs size of 50 nm. (a) Effect of IPD in the existence of the additional resonance, (b) Multipole decomposition analysis polariton branches. The dashed line indicates the position of the resonance and the arrow points to the dominant mode.

**Supplementary Note 6. Particle size distribution**

The particle size distribution is determined from the analysis of scanning electron microscopy (SEM) image using the free software ImageJ. This size distribution contributed to the broadened linewidth of the experimental hybrid polariton branches relative to the calculated optical spectra obtained from simulations.


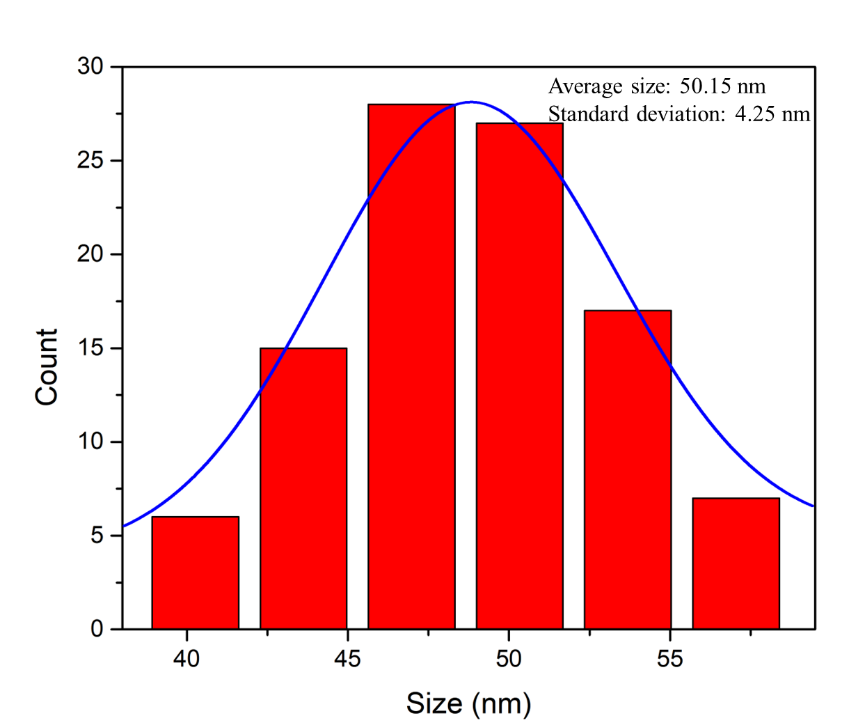


**Figure S9.** Au-NPs size distribution by analysis of SEM image using ImageJ software. The blue line shows the Gaussian fitting.
